# Validation of a computational chain from PET Monte Carlo simulations to reconstructed images

**DOI:** 10.1016/j.heliyon.2022.e09316

**Published:** 2022-04-21

**Authors:** Philip Kalaitzidis, Johan Gustafsson, Cecilia Hindorf, Michael Ljungberg

**Affiliations:** a Medical Radiation Physics, Lund, Lund University, Lund, Sweden; b Department of Medical Radiation Physics and Nuclear Medicine, Karolinska University Hospital, Solna, Stockholm, Sweden

**Keywords:** CASToR, Gate, Monte Carlo simulation, Positron emission tomography, Tomographic reconstruction

## Abstract

The study aimed to create a pipeline from Monte Carlo simulated projections of a Gate PET system to reconstructed images. The PET system was modelled after the GE Discovery MI (DMI) PET/CT, and the simulated projections were reconstructed with the stand-alone reconstruction software CASToR. Attenuation correction, normalisation calibration, random estimation, and scatter estimation for the simulations were computed with in-house programs. The pipeline was compared in both projection and image space with data acquired on a clinical DMI and reconstructed with GE's off-line PET reconstruction software (PET Toolbox) and CASToR. The simulated and measured data were compared for the number of prompt coincidences, scatter fraction, contrast recovery coefficient (CRC), signal-to-noise ratio (SNR), background variability, residual lung error, and image profiles. A slight discrepancy was noted in the projection space, but good agreements were generally achieved in image space between simulated and measured data. The CRC was found to be 81 % for Gate – CASToR, 84 % for GE – CASToR, and 84 % for GE - PET Toolbox for the largest sphere of the NEMA image quality (IQ) phantom, and the SNR was found to be 98 for Gate – CASToR, 91 for GE – CASToR, and 93 for GE – PET Toolbox. Profiles drawn over the spheres for the NEMA IQ phantom and the Data Spectrum (DS) phantom show a good match between measurement and simulation. The results indicate feasibility to utilise the pipeline as a tool for off-line simulation-based studies. A complete pipeline introduces possibilities to study the impact of single parameters in the whole chain from simulation to reconstructed images.

## 1 Introduction

Monte Carlo (MC) simulations are useful for the design, evaluation, and optimisation of positron emission tomography (PET) systems. This, in turn, allows for the improvement of PET-based studies of physiological and biochemical processes in, e.g., oncology, cardiology, and neurology [1, 2, 3, 4]. Improvement of the visual quality and quantitative accuracy of PET images through direct experimentation can be difficult due to the large number of degrees of freedom that affects the result. MC simulations may be helpful in this context since the PET system can be replicated *in silico*, and hard-to-adjust parameters can easily be changed and the effects of the adjustments evaluated. One MC software that is specifically designed for simulations of medical physics devices is Gate [5], an open-source MC application based on the Geant4 toolkit [6]. Gate is able to simulate complex detector systems and geometries, and there have been several Gate simulation studies modelling PET systems [7, 8, 9].

MC simulations alone can be used to extract some parameters of interest for optimisation of the PET system. However, such information will always be an indirect measure of the optimality of system design and acquisition settings. It may be difficult to foresee how a difference in projection space will propagate to the reconstructed image and, in the long term, affect the suitability of the image for a given task. The lack of possibility to evaluate the final product, i.e., the reconstructed image, limits the range of situations in which the MC simulations alone can be used.

In contrast, the combination of MC simulated projections and an off-line reconstruction software allows for a complete simulation-based optimisation and evaluation of acquisition protocols and image processing methods. The combination of MC simulated data and off-line reconstruction has previously been done to some extent for a few simulated and clinical PET scanners [10, 11, 12]. However, we believe there is value in a more extensive validation of the whole simulation-reconstruction chain, evaluating the MC simulated PET model by connecting experimental data to a stand-alone reconstruction software in the sense of how well the PET-image from the pipeline matches the corresponding image from a clinical system.

In the current study, we use the Customizable Advanced Software for Tomographic Reconstruction (CASToR) [13] software, which is an open-source transmission and emission reconstruction framework, that can handle data obtained both from clinical tomographs of different vendors and from data acquired through simulations. The aim of this study was twofold: a) To validate the Gate MC model of GE's Discovery MI (DMI) PET/CT system (GE Healthcare, Chicago, Il, USA) through comparisons of results from phantom experiments and simulations. b) To evaluate the possibility to substitute GE's proprietary reconstruction software with the CASToR software. As it is virtually impossible to import simulated data to the clinical reconstruction workstations, the construction of a simulation-reconstruction pipeline is highly beneficial as it provides possibilities to study the impact of the whole chain for a single parameter as opposed to only evaluating the effects in projection space. The combination of Gate and CASToR completes the pipeline from simulated data to reconstructed images, allowing for further simulation studies to be conducted.

## 2 Materials and methods

Three sets of phantom experiments using ^18^F-Fluorodeoxyglucose (FDG) were performed on a four-ring GE DMI PET/CT with a 20 cm axial field-of-view (FOV).

The first phantom was the Philips PET/CT Uniform Cylinder Phantom (volume 9.0 L) filled with water, which was used to tune parameters of the Gate digitizer with respect to detection efficiency and noise module by comparison of the singles count rate between measurement and simulation. The cylindrical compartment was filled with a total activity of 157.2 MBq (start of acquisition), and projections were acquired for 60 s every 30 min over a total acquisition period of 19 h.

The second phantom was the National Electric Manufacturers Association (NEMA) Image Quality (IQ) phantom (volume 9.7 L) filled with water. The cylindrical Styrofoam lung insert was placed centrally in the phantom, and six spheres with inner diameters 37 mm, 28 mm, 22 mm, 17 mm, 13 mm, and 10 mm were used in a set of two measurements. In the first measurement, the spheres were filled with an activity concentration of 44.8 kBq/mL (start of acquisition) with a sphere-to-background ratio (SBR) of 1:0 (i.e., a non-radioactive background). Projections were acquired during 10 min for a single bed position with the data stored in list-mode format. In the second measurement, the spheres contained an activity concentration of 32.6 kBq/mL (start of acquisition) and the background activity concentration was 3.7 kBq/mL, resulting in an SBR of approximately 9:1. The camera acquisition settings were the same as for the measurement with the cold background.

The third phantom was the Data Spectrum (DS) 11 cm radius cylindrical phantom (volume 6.7 L) filled with water. A secondary set of six spheres were used with the DS phantom with inner diameters 15.43 mm, 12.43 mm, 9.89 mm, 6.23 mm, 4.95 mm, and 3.95 mm. The phantom was imaged twice with the same acquisition parameters as for the second phantom. The spheres were filled with an activity concentration of 26.3 kBq/mL (SBR 1:0) in the first measurement. In the second measurement, the activity concentration in the spheres was 20.7 kBq/mL and the activity concentration in the background (volume 6.4 L) was 2.6 kBq/mL (SBR 8:1).

The four different set-ups from the second and third sets of phantom experiments will henceforth be referred to as

⁃ NEMA-B: NEMA IQ phantom with activity in the background.
⁃ NEMA: NEMA IQ phantom without activity in the background.
⁃ CYL-B: DS phantom with activity in the background.
⁃ CYL: DS phantom without activity in the background.

### 2.1 Extraction of clinical data and reconstruction

After acquisition on the clinical scanner, data were exported from the camera system to the Duetto v02.07 PET Toolbox (GE Healthcare, Chicago, Il, USA) for further processing. PET Toolbox is a package of MATLAB code for off-line reconstruction and data processing of data acquired on a clinical GE PET scanner. The implementations of reconstruction algorithms in the PET Toolbox are equivalent to the implementations on GE's PET scanners. The PET Toolbox was used to extract the list-mode event-by-event information from the experimental acquisition, compute attenuation, normalisation, deadtime, and pile-up correction factors, estimate random and scattered coincidences, and reconstruct the measured data.

#### 2.1.1 Transferring scanner data

The PET list-mode data acquired on a DMI PET/CT is stored in an HDF5 format along with header information of acquisition parameters, and the list-file is automatically compressed on the scanner. The file was decompressed on the scanner with GE's proprietary decompression algorithm before transferral. Additional files for attenuation correction, normalisation, and well-counter calibration were also transferred from the scanner to the PET Toolbox.

#### 2.1.2 Extracting coincidences and defining reconstruction parameters

Each event in the PET list-file contains information of the axial and transaxial crystal indices of the two unique crystals constituting the recorded line-of-response (LOR) and the time-of-flight (TOF) information of that event. The acquired list-mode data were rebinned into sinograms in the PET Toolbox with the UnlistMain() function for the whole 10 min acquisition. A data structure with default user-definable parameters was initialised with the ptbUserConfig() function, from which modifiable parameters for the reconstruction were set.

#### 2.1.3 Reconstruction with PET toolbox

The reconstruction with the PET Toolbox was performed by passing the user-defined data structure to the PET Toolbox reconstruction module, ptbRunRecon(). In the reconstruction module, correction sinograms for scatter, randoms, deadtime, pile-up, and normalisation were computed and saved. TOF sinograms are memory intensive; the TOF scatter sinograms were stored in a down-sampled format and up-sampled with the ptbUpSampleTofScatterSinogram() function.

#### 2.1.4 Reconstruction with CASToR

The GE list-mode data and the associated correction files computed with the PET Toolbox were reformatted into CASToR's list-mode format with an in-house conversion program. In this instance, all available correction data from the PET Toolbox were used (attenuation, normalisation, scatter, random, deadtime, and pile-up correction). As attenuation and normalisation correction factors were applied in the CASToR reconstruction process (i.e., in the system matrix), it was required that these correction factors were considered in the sensitivity image so that the sensitivity image is in agreement with the back-projected image. Therefore, an additional CASToR normalisation file containing the attenuation and normalisation correction factors for every possible LOR was constructed and provided to the reconstruction program for the computation of the sensitivity image. CASToR events are associated with a physical scanner geometry; the geometrical scanner information was defined in a text file that was retrieved by the reconstruction program to compute the physical geometry of the scanner. The geometrical information of the DMI scanner was obtained from the header part of the GE PET HDF5 list-file.

### 2.2 Gate Monte Carlo simulations

In this study, version 8.2 of Gate was used [5]. The physics models for photon and charged-particle interactions were set as the emstandard_opt3 [14] physics list. Production cuts, below which no secondary particles are generated, were set to 1 mm for all volumes.

#### 2.2.1 Simulation geometry and acquisition set-up

The DMI PET scanner model was constructed based on Gate's cylindricalPET-system and consisted of 34 detection modules (rsectors) arranged in a ring, with each detection module comprised of four axial units (modules). A unit contained four transaxial detection blocks (submodules), with each detection block containing an array of 4×9 transaxial by axial lutetium-yttrium oxyorthosilicate (LYSO) scintillating crystals, resulting in a total of 19584 LYSO crystals of dimensions 5.3×3.95×25 mm^3^. The detection modules were arranged with the first module placed directly above the scanner isocenter and with the module number increasing clockwise looking from the front of the gantry. The crystal face-to-face diameter was set to 744.2 mm, and an annulus plastic bore liner with a thickness of 2 mm located 350 mm from the centre of the scanner was also included in the simulation geometry.

#### 2.2.2 Digitizer module

The behaviour of the front-end electronics was simulated in Gate using the digitizer module that replicates the camera system's signal processing chain. The digitizer module processes the information generated between particles and designated volumes to build physical observables from hit snapshots, i.e., information regarding, e.g., energy, position, and timing. At the end of the signal chain, the output event is called a single and the digitizer handles the coincidence logic by constructing coincidences from the single events.

In the case of modelling the signal chain of the DMI scanner, the digitizer should replicate the behaviour of the GE Lightburst digital detector [15] that uses silicon photomultiplier (SiPM) devices to identify the scintillating crystal based on the centroid of relative signals. In the clinical DMI scanner, each detection block is coupled to a Lightburst detector device. The Lightburst digital detector includes the Compton Scatter Recovery (CSR) [16] function, which recovers singles from photons scattered between adjacent-lying detection blocks.

In the current study, the digitizer module was set up in the following order: first, the adder module was included to sum multiple hits within a designated volume, i.e., acting as an integrator of the deposited energy to regroup hits into pulses. The adder was followed by the readout module to regroup pulses within a larger sensitive volume than the smallest single output component (crystal). Two readout module policies exist in Gate, takeEnergyWinner and takeEnergyCentroid. The takeEnergyWinner policy will assign the crystal location by the hit depositing the largest amount of energy within the chosen readout volume, while for the takeEnergyCentroid, the position is determined by weighting the energy pulse from each crystal indices to get the energy centroid position. The readout volume was set to the detection module (rsector) with the policy takeEnergyWinner, as the takeEnergyCentroid policy forces the readout volume to be the level above the crystal volume. Setting the readout volume to the detection module rather than the detection block, to some extent, imitates the behaviour of CSR, even if explicit CSR functionality is currently not implemented in Gate. The noise module was defined after the readout module to simulate the radioactive decay of ^176^Lu inherent in the LYSO crystals. A Gaussian energy blurring was applied to the integrated energy with a full width at half maximum (FWHM) of 9.63 % [17] at 511 keV. A detection efficiency was then defined to account for crystal transfer efficiency and the SiPM quantum efficiency. The parameters used for the background noise frequency and detection efficiency was optimised according to the method described by Guez et al. [18], using the Philips PET/CT Uniform Cylinder Phantom. Additionally, a Gaussian temporal resolution with a FWHM of 268.7 ps to the timing precision was applied to each single to match the DMI coincidence timing resolution of 380 ps. Low and high energy photons were rejected by applying an energy discriminator, the lower threshold was set to 425 keV and the upper level was set to 650 keV.

**Figure 1:**
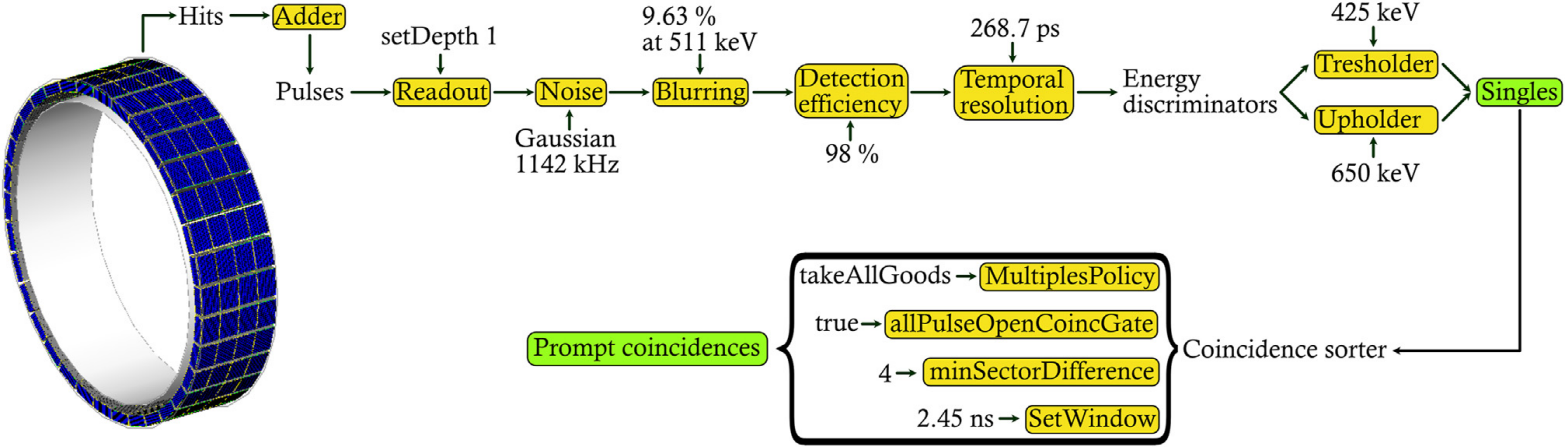
Interactions occurring within the crystals are referred to as hits. For each hit, physical observables are generated, and the hits are integrated and regrouped into pulses. The pulses are affected and constrained by several digitizer modules, which at the end of the digitizer chain single events are created. Coincidences are then constructed from pairs of single events, where, similarly, several digitizer modules are introduced to mimic the coincidence logic of a clinical DMI scanner.

Coincidences were formed from singles using multi-window (MW) mode with a total coincidence timing window (2τ) of 4.9 ns, i.e., the window opened in Gate by a photon interaction (τ) was set to 2.45 ns. Information regarding the coincidence timing window was obtained from the four-ring DMI configuration function in the PET Toolbox. A multiples policy of takeAllGoods was chosen to best mimic the processing behaviour of multiple coincidences for the current PET system [19]. A minimum sector difference of 4 was applied to allow coincidences with a transaxial FOV larger than 700 mm to be recorded. The coincidences were later binned based solely on the coincidence's axial and transaxial crystal indices, at that stage restricting the formation of coincidences to a transaxial FOV of 700 mm. The effects of deadtime and pile-up were not included in the model. The Gate scanner geometry and a schematic overview of the digitization process with the parameters used in the digitizer are shown in Figure 1.

#### 2.2.3 Simulations

Two separate sets of simulations of the Philips PET/CT Uniform Cylinder Phantom were performed to mimic the first measurement. Similar to the first measurement, the simulations were performed for 60 s with the start activity 157.2 MBq, every subsequent 30 min time frame was simulated with the activity adjusted for physical decay accordingly. The background noise frequency used in the simulation was found by extrapolating the measured singles count rate down to zero activity. The first set of simulations was performed without modelling any detection efficiency. Given complete modelling of the Gate digitizer (including deadtime and pile-up), the ratio between measured and simulated singles count rate should be constant over the investigated activity range. However, as deadtime and pile-up were not included in the model, the measured singles count rate for each time frame was adjusted with the singles event deadtime information acquired from the GE PET raw file to circumvent that deadtime and pile-up had not been included in the Gate model. The ratio between measured and simulated singles count rate after the adjustment was used as the system's detection efficiency. The second set of simulations was performed with the inclusion of the determined detection efficiency.

Four additional simulations were performed; two with the NEMA IQ phantom and two with the DS phantom (NEMA-B, NEMA, CYL, and CYL-B). The activity concentration for each simulation was set to match the activity concentration used in the scanner measurements.

**Figure 2:**
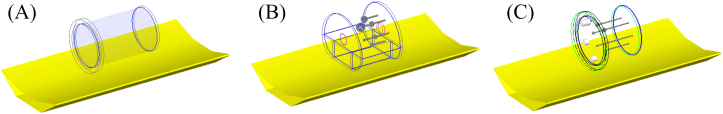
The simulated phantoms, the Philips PET/CT Uniform Cylinder Phantom (A), the NEMA IQ Phantom (B), and the DS Phantom (C), are shown placed on a carbon fibre couch.

The sources used in the simulations were modelled as $\beta^{+}$-particles originating from the decay of ^18^F, with a parameterised $\beta^{+}$ energy-spectrum and emission yield according to the Landolt-Börnstein tables [5]. The simulated phantoms are shown in Figure 2.

#### 2.2.4 Processing simulation data

Each detected single and coincident event was stored in a ROOT [20] list-file format, containing the information regarding interaction volumes, deposited energy, and timestamp for each event. The ROOT file was processed through in-house programs to extract and bin prompt sinograms and estimate random coincidences (section 2.3). The formation of sinograms was determined solely from the axial and transaxial crystal indices of the event. The axial crystal index was determined according to

(1) $$\begin{array}{l} ID_{\text{cry},\text{ax}}=\frac{ID_{\text{rsec}}}{N_{\text{rsec}}}\cdot \left(N_{\text{mod},\text{ax}}\cdot N_{\text{smod},\text{ax}}\cdot N_{\text{cry},\text{ax}}\right)\\ +\frac{ID_{\text{mod}}}{N_{\text{mod},\text{tax}}}\cdot \left(N_{\text{smod},\text{ax}}\cdot N_{\text{cry},\text{ax}}\right)+\frac{ID_{\text{smod}}}{N_{\text{smod},\text{tax}}}\cdot \left(N_{\text{cry},\text{ax}}\right)+\frac{ID_{\text{cry}}}{N_{\text{cry},\text{tax}}},\; \end{array}$$

where

⁃ $ID_{\text{rsec}}$ is the ROOT metadata of the Gate rsector volume ID.
⁃ $N_{\text{rsec}}$ is the number of rsectors.
⁃ $N_{\text{mod},\text{ax}}$ is the number of axial modules in an rsector.
⁃ $N_{\text{smod},\text{ax}}$ is the number of axial submodules within a module.
⁃ $N_{\text{cry},\text{ax}}$ is the number of axial crystals in the submodule.
⁃ $ID_{\text{mod}}$ is the ROOT metadata of the Gate module volume ID.
⁃ $N_{\text{mod},\text{tax}}$ is the number of transaxial modules in an rsector.
⁃ $ID_{\text{smod}}$ is the ROOT metadata of the Gate submodule volume ID.
⁃ $N_{\text{smod},\text{tax}}$ is the number of transaxial submodules in a module.
⁃ $ID_{\text{cry}}$ is the ROOT metadata of the Gate crystal ID.
⁃ $N_{\text{cry},\text{tax}}$ is the number of transaxial crystals in a submodule.

The transaxial crystal index is computed according to

(2) $$\begin{array}{l} ID_{\text{cry},\text{tax}}=ID_{\text{cry}}\%N_{{\text{N}}_{\text{cry},\text{tax}}}+N_{{\text{cry}}_{\text{smod},\text{tax}}}\cdot \left(ID_{\text{smod}}\%N_{\text{smod},\text{tax}}\right)\\ +N_{{\text{cry}}_{\text{mod},\text{tax}}}\cdot \left(ID_{\text{mod}}\%N_{\text{mod},\text{tax}}\right)+\;N_{{\text{cry}}_{\text{rsec},\text{tax}}}\cdot ID_{\text{rsec}},\; \end{array}$$

where

⁃ $N_{{\text{N}}_{\text{cry},\text{tax}}}$ is the number of transaxial crystals within a submodule.
⁃ $N_{{\text{cry}}_{\text{smod},\text{tax}}}$ is the number of transaxial crystals within a submodule.
⁃ $N_{{\text{cry}}_{\text{mod},\text{tax}}}$ is the number of transaxial crystals within a module.
⁃ $N_{\text{smod},\text{tax}}$ is the number of transaxial submodules within a module.
⁃ $N_{\text{mod},\text{tax}}$ is the number of transaxial modules within an rsector.
⁃ $N_{{\text{cry}}_{\text{rsec},\text{tax}}}$ is the number of transaxial crystals within an rsector.

Each division is performed by integer division, and % represents the modulo operator. For each simulation, the LORs spanning two detector crystals were binned into a sinogram of prompt coincidences according to GE's data organisation standard for the four-ring DMI scanner.

### 2.3 Computation of simulated PET data corrections

This section describes the computation of the PET corrections for the tomographic reconstruction of the simulated data. The indices *u*, *i*, *v*, and *j* denote the combination of axial and transaxial detector crystals, e.g., *ui* would be the axial and transaxial crystal index for crystal 1, and *vj* would be the axial and transaxial index for crystal 2, together forming the LOR_uivj_.

#### 2.3.1 Attenuation correction

To correct for attenuation, an image of linear attenuation coefficients at 511 keV of the different materials in the simulated phantom was forward-projected using the Siddon projection algorithm [21]. The attenuation correction factor (*acf*_uivj_) was then computed for all valid LORs according to,

(3) $$acf_{\text{uivj}}=\frac{1}{\exp\left(-\int_{D_{\text{vj}}}^{D_{\text{ui}}}\mu \left(x\right)dx\right)},$$

where *D*_ui_ and *D*_vj_ are the coordinates of the crystal detector elements between which the LOR spanned, and the detector coordinates were set according to the DMI crystal layout.

#### 2.3.2 Normalisation

To correct for non-uniform sensitivity between detector channels, a component-based normalisation [22] approach was implemented according to the method described in [23]. The normalisation correction factor for each crystal pair was computed as

(4) $$\begin{array}{l} \eta_{\text{uivj}}=\varepsilon_{\text{ui}}\;\varepsilon_{\text{vj}}\;b_{\text{u}}^{\text{ax}}\;b_{\text{v}}^{\text{ax}}\;g_{\text{uv}}^{\text{ax}}\;r_{\text{r}}^{\text{tr}}\;f_{\text{φ\%D}}^{\text{tr}},\; \end{array}$$

where *ε* is the intrinsic detector efficiency, *b* is the axial block profile factors, *g* is the axial geometric factor, *r* is the radial profile factor, and *f* is the crystal interference function. To calculate the normalisation factor, *η*_uivj_, two simulations were performed. The first simulation was performed with a cylindrical source placed centrally in the scanner to compute the axial normalisation factors and the intrinsic detector efficiencies, and for the second simulation, an annulus source exceeding the transaxial FOV was used to compute the transaxial normalisation factors. Both simulations were performed with a 2-h acquisition containing an activity of 5 MBq.

#### 2.3.3 Randoms correction

To correct for random coincidences, the number of random events per LOR (*R*_uivj_) were estimated from the singles count in each crystal [24] according to

(5) $$\begin{array}{l} R_{\text{uivj}}=\frac{2\tau S_{\text{ui}}S_{\text{vj}}}{T}, \end{array}$$

where *S*_ui_ and *S*_vj_ are the number of singles measured on the two detector crystals spanning the LOR_uivj_, 2τ is the full coincidence timing window, and *T* is the time duration of the simulations. The crystal identification of *ui* and *vj* was determined from the ROOT metadata according to Eqs. (1) and (2).

#### 2.3.4 Scatter correction

**C**orrection for scattered coincidences was computed through an in-house implementation of the Single Scatter Simulation (SSS) technique [25, 26, 27]. The SSS was performed to estimate the scatter contribution to a LOR_uivj_ by computing the summed contribution to that LOR from all sampled scatter points, *S*. The scatter contribution from a scatter point to a particular LOR_uivj_ is dependent on two individual contributions, i.e., $R_{\text{sct},\text{s}}^{\text{uivj}}=R_{\text{sct},\text{s}}^{\text{ui}}+R_{\text{sct},\text{s}}^{\text{vj}}$. The term $R_{\text{sct},\text{s}}^{\text{ui}}$ contributes to the scatter rate for emitters from scatter point *S* to detector *ui* and $R_{\text{sct},\text{s}}^{\text{vj}}$ is the corresponding contribution from *S* to detector *vj*. $R_{\text{sct},\text{s}}^{\text{ui}}$ and $R_{\text{sct},\text{s}}^{\text{vj}}$ were calculated as

(6) $$\begin{array}{l} R_{\text{sct},\text{s}}^{\text{ui}}=\int_{S}^{ui}\lambda \left(s\right)ds\cdot \exp\left(-\int_{S}^{ui}\mu \left(E,s\right)ds\right)\cdot \exp\left(-\int_{S}^{vj}\mu \left(E^{\prime },s\right)ds\right)\cdot \varepsilon {\left(E\right)}_{\text{ui},\text{s}}\varepsilon {\left(E^{\prime }\right)}_{\text{vj},\text{s}}\cdot \frac{\sigma_{\text{ui}}\sigma_{\text{vj}}}{4\pi l_{\text{s},\text{ui}}^{2}l_{\text{s},\text{vj}}^{2}}\cdot \frac{\mu }{\sigma_{\text{c}}}\frac{d\sigma \left(\theta \right)}{d\text{Ω}}\;,\; \end{array}$$

and

(7) $$\begin{array}{l} R_{\text{sct},\text{s}}^{\text{vj}}=\int_{S}^{vj}\lambda \left(s\right)ds\cdot \exp\left(-\int_{S}^{vj}\mu \left(E,s\right)ds\right)\cdot \exp\left(-\int_{S}^{ui}\mu \left(E^{\prime },s\right)ds\right)\cdot \varepsilon {\left(E\right)}_{\text{vj},\text{s}}\varepsilon {\left(E^{\prime }\right)}_{\text{ui},\text{s}}\cdot \frac{\sigma_{\text{ui}}\sigma_{\text{vj}}}{4\pi l_{\text{s},\text{ui}}^{2}l_{\text{s},\text{vj}}^{2}}\cdot \frac{\mu }{\sigma_{\text{c}}}\frac{d\sigma \left(\theta \right)}{d\text{Ω}}\;.\; \end{array}$$

*E* is the unscattered photon's energy, *E′* is the photon energy after Compton scatter, λ(s) is the source distribution of the object, μ(E,s) is the linear attenuation coefficient. $\varepsilon_{\text{ui},\text{s}}$ and $\varepsilon_{\text{vj},\text{s}}$ are the detector efficiencies, $\sigma_{\text{ui}}$ and $\sigma_{\text{vj}}$ are the geometric cross-sections of the detectors presented normally to the rays $\bar{Sui}$ and $\bar{Svj}$, *l*_s,ui_ and *l*_s,vj_ are the distances between the scatter point and the detectors *ui* and *vj*. $\sigma_{\text{c}}$ is the total Compton interaction cross-section, and dσ(θ)/dΩ is the differential scattering cross-section for a photon scatter by an angle *θ* into the solid angle *dΩ*.

**Figure 3:**
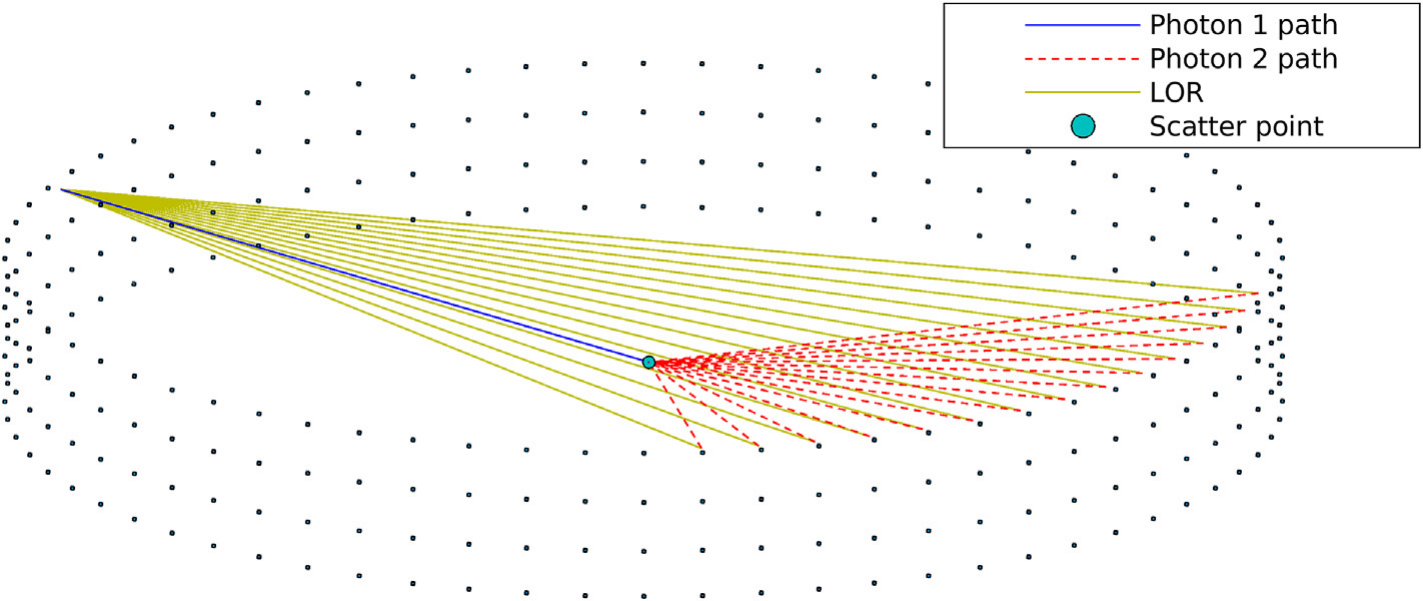
A down-sampled detector system where the scatter point has been sampled inside the phantom. One of the two photons is directed towards a detector from the scatter point without scattering, and the other photon is directed towards another detector with reduced energy after scattering at the sample point. A few possible LORs are shown for that given scatter point and unscattered photon trajectory.

The initial source distribution image was reconstructed using filtered back projection, corrected for randoms, attenuation, and normalisation. Both the attenuation and source distribution images were down-sampled to a 32×32×4 grid, speeding up the process of computing the ray-sums in Eqs. (6) and (7). The down-sampled attenuation image was also used to sample scatter points, rejecting samples with a linear attenuation coefficient less than 0.04 cm^−1^. A random spatial offset was introduced within each selected scatter point to avoid the occurrence of artefacts [26, 28]. Additionally, the detector system was down-sampled to 68 transaxial and 4 axial crystals to achieve feasible computation times. For each scatter point, *S*, all possible crystal pairs that yields a valid LOR was determined, for which Eqs. (6) and (7) were computed. The total scatter coincidence rate for a particular LOR_uivj_ was then expressed as the summation of scatter contributions from all sampled scatter points in the object, i.e.,

(8) $$\begin{array}{l} R_{\text{sct},\text{tot}}^{\text{uivj}}=\sum_{s}^{N}R_{\text{sct},\text{s}}^{\text{uivj}}, \end{array}$$

where $R_{\text{sct},\text{tot}}^{\text{uivj}}$ represents the total scatter estimate after evaluating all sampled scatter points. The SSS was extended to handle TOF-dependent scatter estimation according to [29]. Figure 3 shows an example of the SSS process from which a few possible LORs have been formed between the unscattered and the scattered photons.

After all sample points had been evaluated, the estimated scatter sinogram was up-sampled to the original sinogram size using cubic splines in radial and axial directions and linear interpolation in the azimuthal direction. The estimated scatter sinogram, *s*, was then scaled to the full sinogram, *y*, with a least-square fit, $\underset{\text{b}}{\sum }w_{\text{b}}{\left(y_{\text{b}}-s_{\text{b}}\beta -q\right)}^{2}$, for the determination of scale factors *β* and *q*, performed over the region containing no unscattered events. The intercept, *q*, was set to adopt the value 0 for isotopes that do not emit prompt gamma. A weighting factor, *w*, was introduced and set to 1 for the bins *b* within the scatter-only region and 0 elsewhere. The tails of the sinogram (scatter-only region) were found by forward projecting the attenuation image. The SSS was performed iteratively to estimate the scattered coincidences. The convergence was sped up by averaging the estimated scatter contribution of the two first iterations [30].

#### 2.3.5 Well-counter calibration

A well-counter calibration was performed in Gate to convert the reconstructed image from arbitrary units to absolute activity concentration. A 20 min simulation was performed using a cylindrical phantom with a radius of 9.5 cm and a length of 20 cm. Tomographic images were reconstructed using CASToR, including all previously described corrections. The calibration factor was computed by placing a region of interest (ROI) in each transverse slice of the reconstructed object. With a known activity concentration of 4.4 kBq/mL, the well-counter calibration value was extracted as

(9) $$\begin{array}{l} WCC=\frac{C_{\text{abs}}}{C_{\text{arb}}}, \end{array}$$

where *C*_abs_ is the activity concentration in the phantom, and *C*_arb_ is the average counts obtained from the ROI in the reconstructed image.

### 2.4 Tomographic reconstruction

The in-house GE-to-CASToR conversion program was extended to convert Gate data and corresponding corrections (normalisation, scatter, randoms, and attenuation) to the CASToR list-mode format.

Scanner data were reconstructed with both the PET Toolbox and CASToR using the Ordered Subsets Expectation-Maximisation algorithm (OS-EM) with 4 iterations and 16 subsets, and the distance-driven projector [31] and the Joseph projector [32], respectively. Corrections obtained from the PET Toolbox (attenuation, randoms, scatter, deadtime and pile-up, and normalisation) were included in scanner data reconstructions with the PET Toolbox and CASToR. Simulated data were reconstructed with CASToR using OS-EM with 4 iterations and 16 subsets and the Joseph projector. Corrections for attenuation, randoms, scatter, and normalisation were included in the reconstruction. The CASToR reconstructed images were post-filtered with a Gaussian kernel with a 4 mm transaxial and 4 mm axial FWHM, and the PET Toolbox reconstructed images were post-filtered with a 4 mm transaxial Gaussian kernel and with GE's heavy z-filter.

### 2.5 Evaluation

The scanner model was evaluated by comparing the measured and simulated sinograms. The number of prompt coincidences between the measurement and simulation was compared, and the scatter fraction (*SF*) was evaluated according to

(10) $$\begin{array}{l} SF=\frac{S}{T+S}, \end{array}$$

where *S* is the number of scattered coincidences, and *T* is the number of true coincidences. The number of true coincidences was estimated by subtracting the number of estimated random and scattered coincidences from the prompt coincidences.

The image qualities were compared according to the NEMA NU 2–2007 standard [33]. Sixty (60) ROIs were placed in the background compartment (NEMA-B and CYL-B) with the mean activity concentration (*B*_i_) and the standard deviation (*σ*_i_) for all voxels within the 60 ROIs were computed. The mean sphere activity concentration (*S*_i_*)* was calculated from a ROI placed over the evaluated sphere. The index *i* signifies the diameters of the ROIs drawn, matching the diameter of the sphere being evaluated. The signal-to-noise ratio (SNR) [34], contrast recovery coefficient (CRC), and background variability (BV) were computed for each sphere for both NEMA-B and CYL-B. The SNR was computed as

(11) $$\begin{array}{l} SNR_{\text{i}}\;=\;\frac{S_{\text{i}}-B_{\text{i}}}{\sigma_{\text{i}}}. \end{array}$$

The CRC between a sphere and the background was computed according to

(12) $$\begin{array}{l} CRC_{\text{i}}\;=\;\left(\frac{\frac{S_{\text{i}}}{B_{\text{i}}}-1}{\frac{a_{\text{H}}}{a_{\text{B}}}-1}\right), \end{array}$$

where $a_{\text{H}}/a_{\text{B}}$ represents the activity SBR.

The BV was calculated as

(13) $$\begin{array}{l} BV_{\text{i}}\;=\;\frac{\sigma_{{\text{BV}}_{\text{i}}}}{B_{\text{i}}}, \end{array}$$

where $\sigma_{{\text{BV}}_{\text{i}}}$ is the standard deviation of the mean of the 60 ROIs with diameter *i*.

The accuracy of attenuation and scatter correction was assessed as the difference between the expected and measured counts in the lung insert as the residual lung error [33, 35], ΔC, for NEMA-B as

(14) $$\begin{array}{l} \Delta C=\frac{C_{\text{Lung}}}{B_{\text{Ø}=37}}, \end{array}$$

where C_Lung_ is the mean activity concentration in a circular ROI with a diameter of 30 mm placed centrally on the lung insert of the NEMA IQ phantom, and B_Ø=37_ is the mean activity concentration of the 60 background ROIs with 37 mm diameter. The residual lung error was taken as an average over 60 slices.

## 3 Results

### 3.1 Sinograms, count rate, and scatter fraction

**Figure 4:**
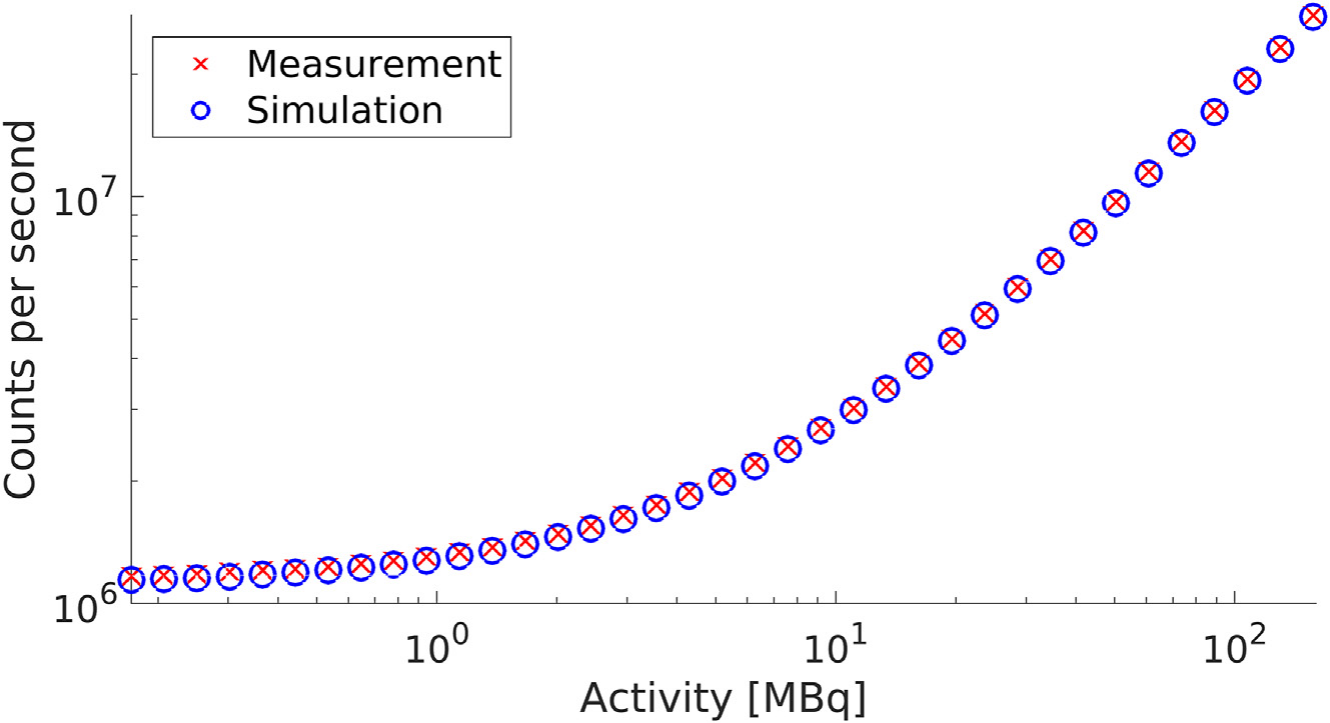
Simulated and measured singles count rate with the Philips PET/CT Uniform Cylinder Phantom, with the measured singles count rate adjusted by the singles event deadtime information acquired from the header in the GE PET raw file.

The measured singles count rate after the singles event deadtime adjustment, and the simulated singles count rate for the second sets of simulations with the Philips PET/CT Uniform Cylinder Phantom are shown in Figure 4. The background noise frequency and the detection efficiency used in the simulations were 1142 kHz and 98 %, respectively.

**Figure 5:**
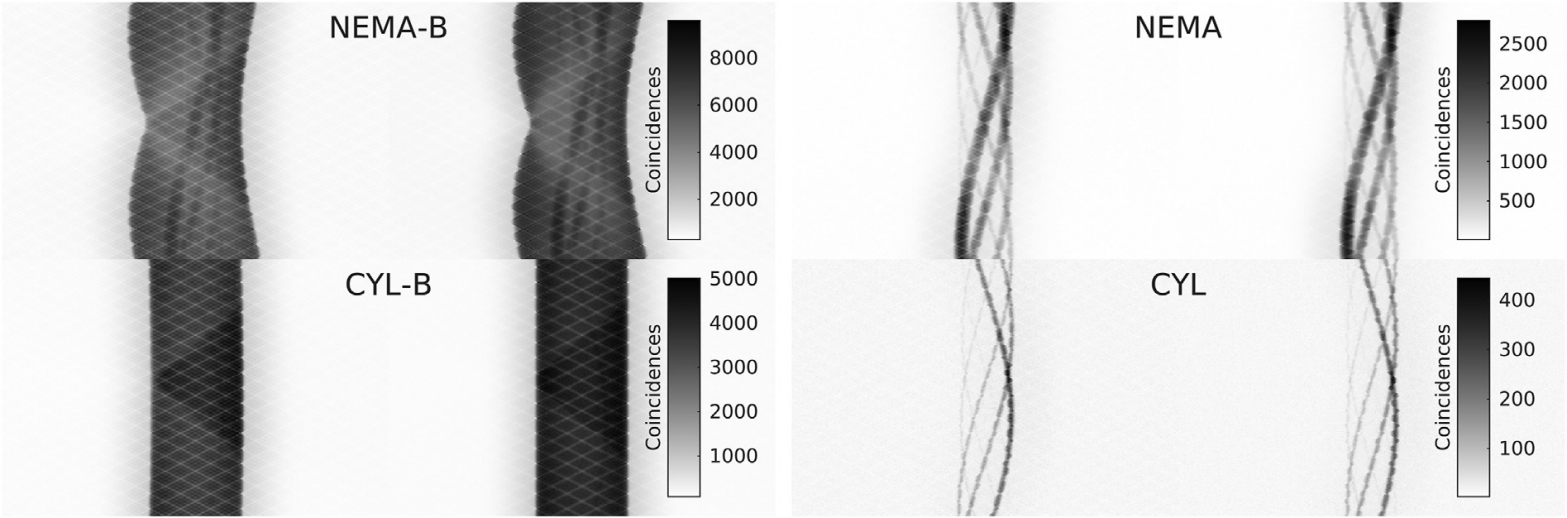
Summed slice of sinogram of prompt coincidences for the measurement (left) and simulation (right). The sinograms are shown with the radial index presented horizontally and the azimuthal index presented vertically.

Prompt sinograms, summed over all axial slices, from the simulation and measurement are shown in Figure 5.

The number of prompt coincidences and scatter fractions are presented in Table 1. The simulated number of prompt coincidences are higher for NEMA-B and CYL-B compared to the prompt coincidences of the corresponding measurements. The difference in the number of prompt coincidences for the low activity measurements and simulations, NEMA and CYL, is lower compared to the differences seen for NEMA-B and CYL-B. The relative difference in the number of prompt coincidences between simulation and measurement amounted to 10.2 % (NEMA-B), 3.1 % (NEMA), 10.2 % (CYL-B), and -2.9 % (CYL).

**Table 1:** Prompt coincidences and scatter fractions from the measurements and simulations.

Parameter	NEMA-B	NEMA	CYL-B	CYL
Prompt coincidences - simulation	2.6 × 10^8^	1.6 × 10^7^	1.3 × 10^8^	2.2 × 10^6^
Prompt coincidences – measurement	2.4 × 10^8^	1.6 × 10^7^	1.2 × 10^8^	2.3 × 10^6^
Scatter fraction – simulation	37.3 %	27.5 %	33.6 %	29.7 %
Scatter fraction – measurement	38.5 %	27.6 %	35.0 %	31.2 %

The largest difference in SF between measurement and simulation was found for CYL with a 1.5 percentage points difference.

### 3.2 Image quality

**Figure 6:**
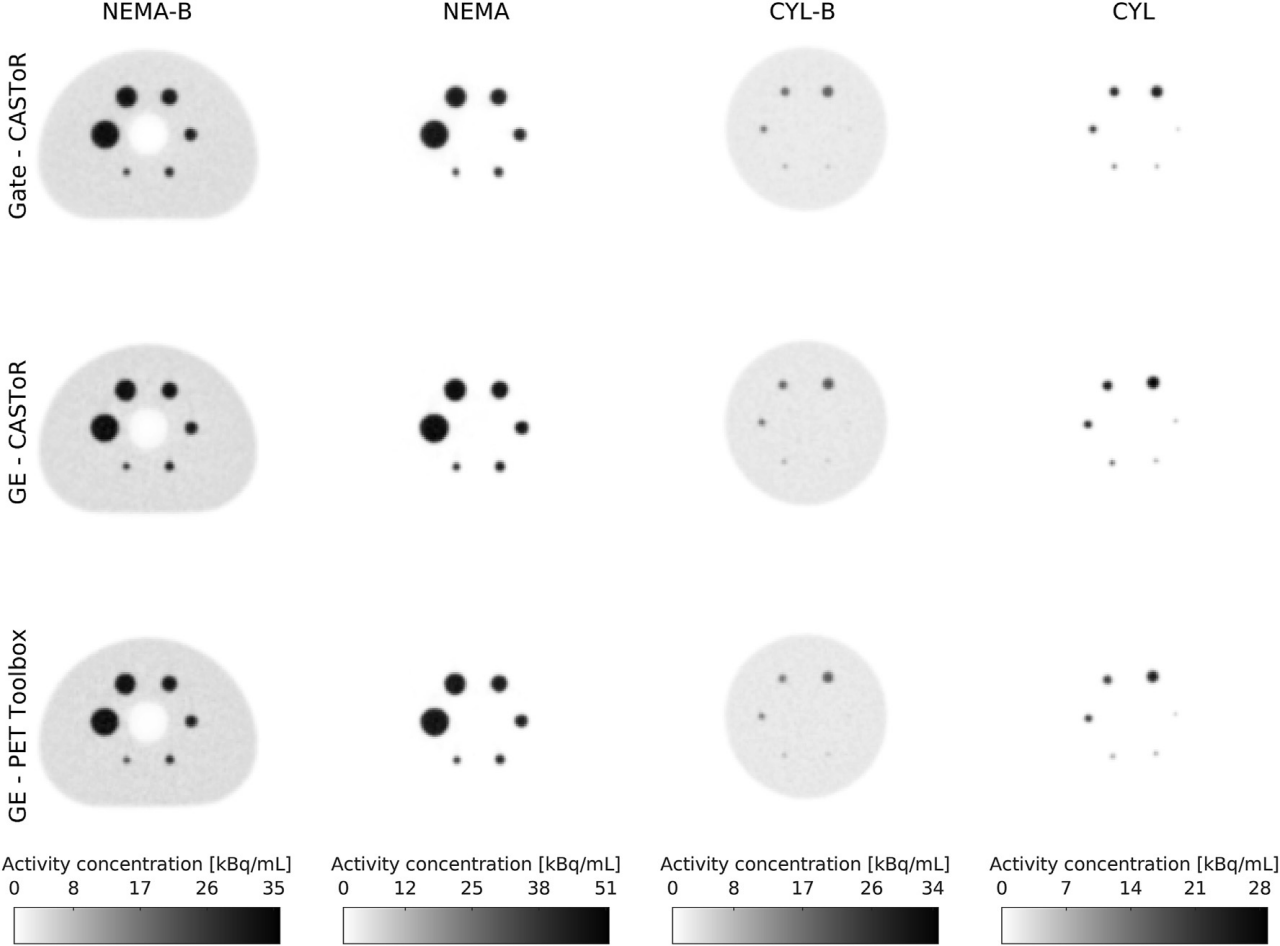
Transversal slices of NEMA-B, NEMA, CYL-B, and CYL. Each column with a certain measurement/simulation is displayed with its own colour scale for representation.

Measured and simulated data reconstructed with the PET Toolbox and CASToR are shown in Figure 6.

The reconstructed images are visually similar and with no major deviations or artefacts. The possibility to distinguish the smallest sphere (Ø = 3.95 mm) for CYL-B and CYL is limited, especially with activity in the background. The second smallest CYL-B sphere is likely to be mistaken for noise, a feature shared regardless of whether the images were reconstructed from measured or simulated data.

**Figure 7:**
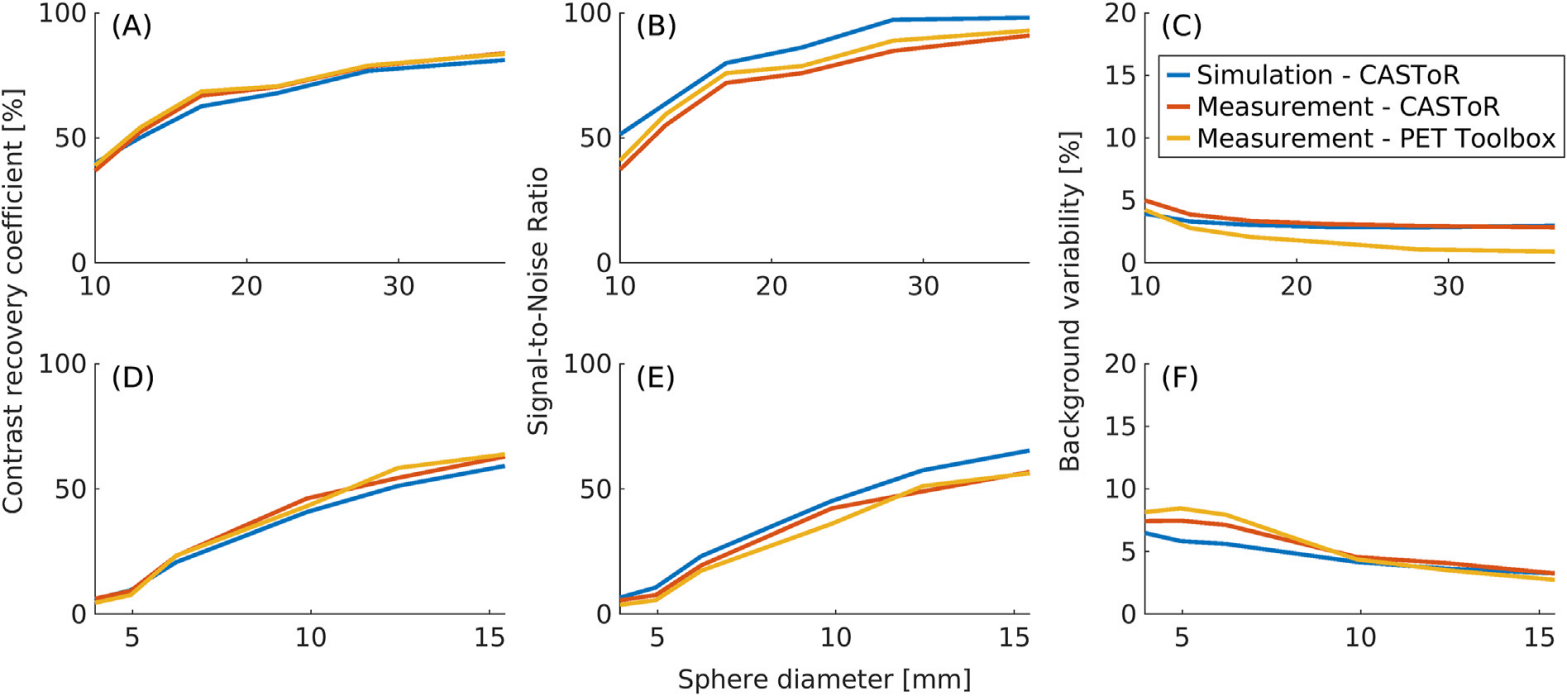
Comparisons of CRC (A and D), SNR (B and E), and BV (C and F) between measured and simulated data for NEMA-B (A–C) and CYL-B (D–F).

Figure 7 shows CRC, SNR, and BV for NEMA-B (A - C) and CYL-B (D - F). The CRC shows good agreement between measurement and simulation for all reconstruction methods. The CRC decreases with decreasing sphere size for both the measured and simulated data, falling below 10 % for the 2 smallest spheres for CYL-B. The SNRs agree well, with a slightly higher SNR for the simulated data than for its measured counterparts. A larger difference between data reconstructed with CASToR and the PET Toolbox for NEMA-B with respect to BV is observed, the difference in BV is more prominent for the larger spheres with a maximum of 2.9 percentage points. CYL-B show a good match in BV for the larger spheres and with a slight deviation for the smaller spheres between the measured and simulated data.

**Figure 8:**
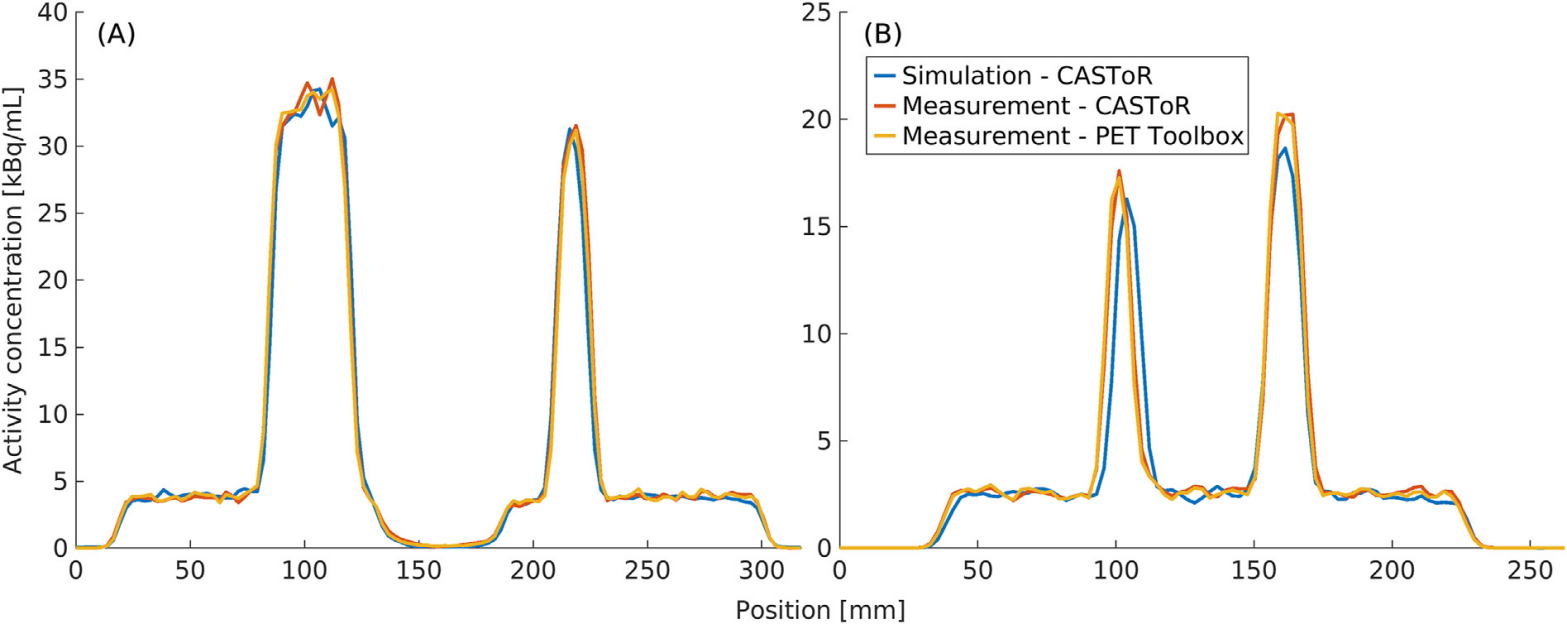
Profiles through NEMA-B (A) and CYL-B (B). The NEMA-B profile (A) runs through the largest (Ø = 37 mm) and the third smallest (Ø = 17 mm) NEMA-B sphere and the CYL-B profile (B) runs through the largest (Ø = 15.43 mm) and the second largest (Ø = 12.43 mm) CYL-B sphere.

Profiles running through the largest and third smallest spheres for NEMA-B and the largest and second largest spheres for CYL-B are shown in the left and right graphs of Figure 8, respectively. The profiles are shape-wise very similar, an indication of similar resolution and activity concentration recovery. For CYL-B, a slightly lower activity concentration was regained in the spheres compared to the measured data, and additionally, the profile conveys a slight misplacement of the simulated left sphere in relation to the measured data.

**Figure 9:**
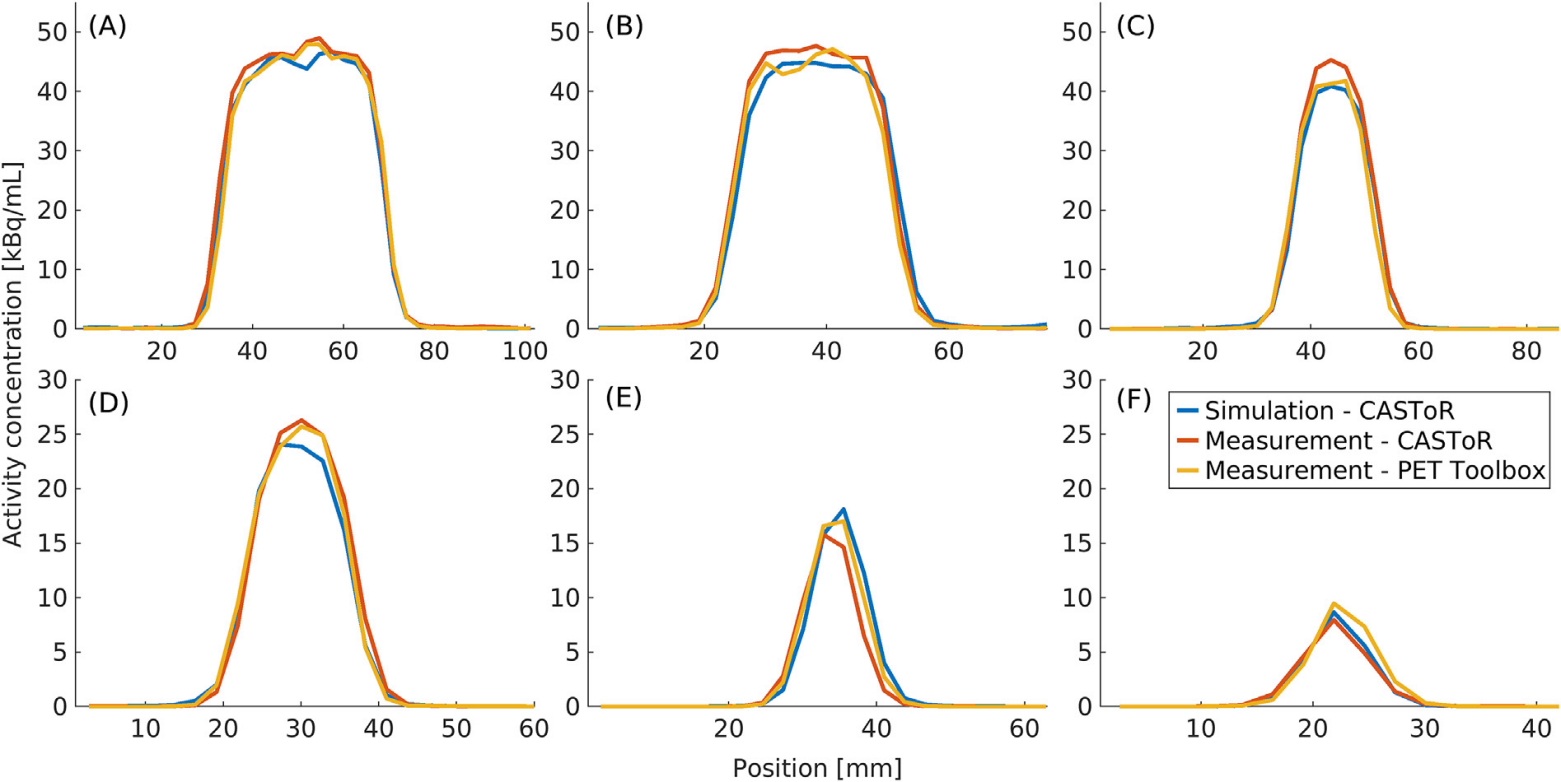
Profiles through NEMA (A–C) and CYL (D–F). The profiles through NEMA (A–C) are shown for the spheres with diameters 37 mm (A), 28 mm (B), and 17 mm (C). The profiles through CYL (D–F) are shown for the spheres with diameters 15.43 mm (D), 9.89 mm (E), and 6.23 mm (F).

Figure 9 show profiles for NEMA and CYL. The NEMA profiles are drawn over the largest (Ø = 37 mm), the second largest (Ø = 28 mm), and the third smallest sphere (Ø = 17 mm), and the CYL profiles are drawn over the largest (Ø = 15.43 mm), third largest (Ø = 9.89 mm), and the third smallest sphere (Ø = 6.23 mm). The profiles have been translated to a common coordinate system.

The average residual lung error amounted to 4 % for the simulated data, 6 % for the measured data reconstructed with CASToR, and 6 % for the measured data reconstructed with the PET Toolbox. The average residual lung error was 2 percentage points lower for the simulated data compared to the measured data.

## 4 Discussion

The combination of Gate MC simulations and tomographic reconstruction with CASToR forms a complete pipeline from simulation to reconstructed images and allows for task-based optimisation of PET imaging using simulations. However, for such optimisation to be relevant, the pipeline must be validated to certify that the obtained results are correct and realistic. We have taken great care to compare the simulated and measured data, and the subsequent reconstruction of said data was compared against data acquired on a clinical DMI scanner, reconstructed with the vendor's reconstruction algorithm and the stand-alone reconstruction software. To accommodate a realistic pipeline from simulation to reconstructed images, the corrections were computed in accordance with methods used on a clinical scanner rather than using simulated metadata, which would yield ideal results, something we cannot hope to achieve on a clinical scanner.

Ideally, the readout volume would be set to the detection block to replicate the same geometrical structure each Lightburst digital detector operates on. However, by choosing the readout level to be at the detection module, the CSR functionality can be imitated, whereas the omission of CSR would lead to a systematic underestimation of the number of prompt coincidences. Instead, by setting the readout level to the detection module, an overestimation of the number of prompt coincidences is expected, as previously shown by Khalifé et al. [8]. With the inclusion of a detection efficiency, crystal light transfer efficiency and SiPM quantum efficiency are accounted for, and thereby aims to compensate for the increased number of recorded prompt coincidences. For the simulations with higher activities, NEMA-B and CYL-B, the discrepancy in the number of prompt coincidences may be explained by the omission of deadtime and pile-up. When determining the detection efficiency, the measured singles count rate was adjusted by the expected singles loss due to deadtime, making the ratio between the measured and simulated singles count rate from the Philips PET/CT Uniform Cylinder Phantom acquisitions constant over the investigated activity range. If not adjusted for, the singles count rate ratio would not be constant over the investigated activity range without modelling deadtime and pile-up. For the activity levels used in NEMA-B and CYL-B, the actual singles count rate ratio between measured and simulated data amounted to approximately 94 % (data not shown) prior to the adjustment of the measured singles count rate, as opposed to the constant 98 % used in the simulations. As the effects of deadtime and pile-up increase with higher activity, more singles would be recorded for these simulations compared to the corresponding measurements, resulting in a larger number of recorded prompt coincidences. The discrepancy in the number of prompt coincidences between the low activity measurements and simulations, NEMA and CYL, is thus expected to be lower as the effects of deadtime and pile-up are smaller. So, while the measured and simulated sinograms in Figure 5 have similar shapes, for both NEMA-B and CYL-B, darker regions can be seen highlighting the higher number of prompt coincidences recorded, which is again seen in Table 1.

For NEMA and CYL, a higher randoms-to-trues ratio (RTR) compared to NEMA-B and CYL-B is recorded. A higher RTR is expected for low activity measurements using scanners with lutetium-based crystals [36], and for corresponding low activity simulations, accurate implementation of the background signal originating from ^176^Lu in the lutetium-based crystals [18, 37] is essential [38]. After investigation of the simulated ROOT metadata, the number of prompt coincidences where at least one of the single events originated from ^176^Lu was found to be 64.2 % for CYL and 13.5 % for NEMA (data not shown), highlighting the importance of accurate modelling of the background signal for very low activity simulations. The slight discrepancy in prompt coincidences seen between the low activity simulations and measurements are generally not an issue in clinical measurements as the activity used is much higher than the background signal originating from ^176^Lu in the LYSO crystals. As the activities used in the NEMA and CYL simulations are well below what is used in routine clinical ^18^F-FDG measurements, e.g. [39, 40], the discrepancy is not expected to be a limitation to the applicability of the model moving towards more clinically realistic situations.

The largest difference in SF between measurement and simulation was found to be 1.5 percentage points for CYL. For NEMA, the estimated SF appeared to deviate to a large extent from NEMA-B for both measurement and simulation, given that the same phantom geometry was used. Therefore, the calculated SF was compared with the SF calculated from the simulated metadata (data not shown) to further investigate the scatter estimation for NEMA. The NEMA simulated SSS estimated SF and the corresponding SF computed from the ROOT metadata differed by 9.5 percentage points, an indication that the SSS estimated scatter contribution was underestimated. Furthermore, looking at the total activity within the image volume for the NEMA measurement, the total activity regained was 33 % higher than the expected 2.1 MBq. The same evaluation was done for CYL, and the activity 0.1 MBq used in the measurement was recovered, an indication that the GE scatter estimation may also have underestimated the scatter contribution for NEMA. For the other SFs the differences are less notable, with the SFs estimated from the ROOT metadata (data not shown) comparable to the SSS estimated SFs. The slight differences in SF between the measurements and the simulations might be explained by omissions of certain modules in the in-house implemented SSS. For example, the current implementation of the in-house SSS does not explicitly model multiple scatters. While multiple scatters are to some degree compensated for in the SSS scaling process, that compensation may not be entirely sufficient [41, 42] in some cases. Therefore, plans to include additional features, such as multiple scatter and scatters originating from outside the axial FOV estimation, have been considered, but for the scope of this study, the current SSS implementation was considered adequate.

The images in Figure 6 are structurally similar with no visible defects or artefacts. Initially, the similarity of the reconstructed images indicates the feasibility of using CASToR as an alternative stand-alone reconstruction software to the vendor's reconstruction workstation. However, as CASToR is generic, there are certain limitations in comparing the results of the PET Toolbox with CASToR, e.g., the implementation of the distance-driven projector [31] is not compatible with compression in the data. Therefore, while the PET Toolbox utilises the distance-driven projector, a different projector had to be chosen for the CASToR reconstructions.

In Figure 7, the CRC for NEMA-B and CYL-B is shown as a function of sphere size, and the results are comparable between measured and simulated data. For the largest NEMA-B sphere the CRC amounted to 81 % for the simulated data and 84 % for the measured data when reconstructed with both CASToR and the PET Toolbox. For the smallest NEMA-B sphere, the CRC amounted to 40 %, 36 %, and 39 % for the simulated data, measured data reconstructed with CASToR, and measured data reconstructed with the PET Toolbox, respectively. Similar CRCs have previously been reported [17, 43]. For CYL-B, the trend is similar, albeit the two smallest spheres (Ø = 4.95 mm and Ø = 3.95 mm) show a CRC of less than 10 %, which is reflected in the visibility of those spheres in Figure 6. The largest CYL-B sphere (Ø = 15.43 mm) shows a slightly lower but comparable CRC with the third smallest NEMA-B sphere (Ø = 17 mm), both in the range of 60–70 %. A similar CRC between measurement and simulation is essential for future studies as it reflects the possibility of distinguishing small lesions.

The SNR, middle column in Figure 7, is shown as a function of sphere size. The low SNR in the two smallest CYL-B spheres poses a problem when attempting to distinguish a signal from noise, with the smallest sphere hard to distinguish at all and the second smallest sphere could potentially be interpreted as noise in Figure 6. The SNR is similar between measurement and simulation, albeit slightly higher for the simulated data. A higher SNR is expected for the simulated data given that the number of prompt coincidences is higher since deadtime and pile-up were not included in the Gate model.

A slight discrepancy in BV between simulated and measured data is noticeable in Figure 7. The most pronounced difference in BV can be seen between data reconstructed with the PET Toolbox and data reconstructed with CASToR for the larger spheres of NEMA-B, with the BV being lower when reconstructed with the PET Toolbox. A difference between the PET Toolbox and CASToR is the type of post-filter; CASToR utilises a Gaussian kernel for transaxial and axial filtering, while a 3-point filter (z-filter) is used axially in the PET Toolbox. The size of the z-filter has been shown to have a larger impact on the BV than the size of the Gaussian filter [44]. Regarding CYL-B, the difference is not as prominent and is more pronounced for the three smallest spheres.

The profiles in Figures 8 and 9 show a good agreement between measured and simulated data. There are some offsets between the measured and simulated data caused by the difficulty of perfectly aligning a simulation set-up to a measurement. Regardless, the good match between the simulated and measured data indicates a similar spatial resolution. Additionally, the profiles show that the pipeline can recover the activity concentration to the same degree as the measured data. The possibility of recovering the activity used in the simulation opens for the feasibility to use the model in studies looking at, e.g., standardised uptake value (SUV).

The average residual lung error has previously been reported for TOF-OSEM reconstruction on the DMI to 7.4 % [45]. For measured data reconstructed with CASToR and the PET Toolbox, the average residual lung error amounted to 6 %, while the average residual lung error was 4 % for the simulation. The simulated residual lung error is lower than the measured, which could be partly explained by the settings of the SSS [46]. In the transaxial down-sampling of the attenuation image, there is a risk that the pixels corresponding to the lung insertion are misrepresented in the coarse sampling, resulting in a slightly different scatter contribution originating from those pixels. Additionally, comparing the Gate and GE attenuation maps, the linear attenuation coefficients used for the simulation were found to be approximately 28 % lower (data not shown) in the lung insert region. The accuracy to which the linear attenuation coefficients are scaled from the acquired CT Hounsfield Units will ultimately affect the accuracy of the PET attenuation correction. Thus, the linear attenuation coefficients for the lung insert material in the measurement may have been overestimated, which would result in a higher activity regained in the lung insert region and would contribute to the difference seen in the average residual lung error between measurement and simulation.

The complete pipeline from simulation to reconstructed image extends the range of uses in which PET MC simulations are beneficial. In clinical situations, where answers to questions may be challenging to study in vivo, the pipeline, combined with anthropomorphic phantoms [47], creates clinically realistic situations that can be studied in silico. For example, the pipeline can be used to set up clinically realistic acquisitions for a range of different patient morphologies. The purpose might be to find an optimal injection activity to spare the patient radiation dose with minimal impact on image quality. Here, the PET MC model allows us to change a single parameter, in this case, injected activity, and the connection to a reconstruction software produces clinically relevant answers, e.g., how lesion detectability or image-noise are affected. The range of uses where the pipeline is beneficial are plentiful, e.g., to study the effect of motion, breathing, or heartbeat, and with the pipeline, these factors can be excluded, something that cannot be done in a clinical situation.

## 5 Conclusion

Completing the Gate model allows for future simulation-based studies of clinically realistic situations. The connection from MC simulated data to CASToR reconstructions opens possibilities to perform complete off-line simulation-based studies. The results from the off-line pipeline are comparable with the results acquired on the clinical scanner, indicating that the pipeline can be used for future PET related studies, e.g., optimisation of acquisition protocols, image quality performance, or evaluation of reconstruction algorithms.

## Declarations

### Author contribution statement

Philip Kalaitzidis, Johan Gustafsson, Cecilia Hindorf, Michael Ljungberg: Conceived and designed the experiments; Performed the experiments; Analyzed and interpreted the data; Contributed reagents, materials, analysis tools or data; Wrote the paper.

### Funding statement

Michael Ljungberg was supported by 10.13039/501100004635Fru Berta Kamprads Stiftelse (FBKS 2017-33 and 2019-44). Philip Kalaitzidis was supported by Kungliga Fysiografiska Sällskapet i Lund (41843).

### Data availability statement

The data that has been used is confidential.

### Declaration of interests statement

The authors declare no conflict of interest.

### Additional information

No additional information is available for this paper.
